# Recurrent Guillain-Barré Syndrome: A Case Report and Literature Review

**DOI:** 10.7759/cureus.94532

**Published:** 2025-10-14

**Authors:** Arush E Michael, Ivy A Sebastian, Sandeep Kaur, Shreya S Philip, Jeyaraj D Pandian

**Affiliations:** 1 Neurology, Christian Medical College, Ludhiana, IND

**Keywords:** autoimmune neuropathy, intravenous immunoglobulin (ivig), landry-guillain-barré syndrome, nerve conduction study (ncs), plasmapheresis, recurrent lgbs

## Abstract

Guillain-Barré syndrome (GBS) is an acute polyradiculoneuropathy, which is generally considered to be monophasic, but recurrences can occur in rare cases. A 66-year-old lady presented with complaints of bilateral lower limb weakness progressing to bilateral upper limb involvement over 20 days. The examination revealed lower limb weakness and absent deep tendon reflexes. She had a history of two similar episodes, diagnosed as GBS, with nerve conduction studies showing demyelinating polyneuropathy and elevated protein in the cerebrospinal fluid. The current episode revealed identical findings, along with positive anti-ganglioside antibodies. She was treated with intravenous immunoglobulin (IVIG) and physiotherapy, resulting in partial recovery at discharge three weeks after symptom onset and near-complete resolution at the three-month follow-up. This case enriches the understanding of recurrent GBS and the importance of differentiating it from treatment-related fluctuation and or chronic inflammatory demyelinating polyneuropathy with acute onset. It further sheds light upon diagnostic interventions and recent advances in management.

## Introduction

Guillain-Barré syndrome (GBS), first described in 1916, is a severe, rapidly progressive, and potentially life-threatening neurological disorder [1]. *Campylobacter jejuni* enteritis is the most frequent antecedent infection incriminated in eliciting the molecular mimicry between bacterial and peripheral-nerve components, with association in 30% of the cases of GBS. The syndrome manifests as a spectrum of peripheral-nerve disorders with several clinical variants that are characterized by the distribution of weakness of the limbs or cranial-nerve-innervated muscles, underlying pathological abnormalities, and associated autoantibodies [2]. The clinical variants encompassed by GBS include acute inflammatory demyelinating polyneuropathy (AIDP) and axonal forms such as acute motor axonal neuropathy (AMAN) and acute motor-sensory axonal neuropathy (AMSAN) [3]. Timely diagnosis and intervention are essential to prevent complications such as respiratory failure and long-term disability.

GBS is usually a monophasic disease; however, it can recur, especially in patients under 30, with milder symptoms, and in those with Miller-Fischer syndrome [4]. Recurrence of GBS is an infrequent phenomenon; recurrences have been reported in approximately two to six percent of patients [5]. A recurrence is defined as two or more episodes that fulfill the National Institute of Neurological and Communicative Diseases and Strokes (NINCDS) criteria for GBS, with a minimum time between episodes of two months (when fully recovered in between) or four months (when only partially recovered) [6]. The cause of recurrence is still uncertain, especially since different preceding events can be present before attacks, and different GBS variants can affect the same patient [7]. However, it is hypothesized that individual immunological differences and potential clustering of autoimmune disorders, combined with genetic host factors, could modulate the well-known antiganglioside antibodies and molecular mimicry mechanisms [6].

Despite being an uncommon entity, recurrent GBS holds clinical significance as it generates diagnostic and therapeutic challenges along with the potential for more severe or peculiar presentations compared to the initial episode. It is essential to differentiate recurrent GBS from two specific entities, namely, chronic inflammatory demyelinating polyneuropathy (CIDP) with acute onset (A-CIDP) and GBS patients with treatment-related fluctuations (GBS-TRF), as it directly impacts therapeutic decisions and long-term management strategies. A-CIDP is defined as a CIDP patient in whom the nadir of the first episode is within eight weeks of onset, and the consecutive course is chronic, as in CIDP [8]. GBS-TRF is defined as (1) improvement in the GBS disability scale of at least one grade or improvement in the Medical Research Council (MRC) sum score more than five points after completion of therapy (2 g of intravenous immunoglobulin (IVIG) per kilogram body weight in two to five days), followed by a worsening of the GBS disability scale of at least one grade or a decrease in the MRC sum score of more than five points within the first two months after the disease onset, or (2) stabilization for more than one week after completion of therapy, followed by a worsening of the GBS disability scale of more than one grade or more than five points on the MRC sum score within the first two months after disease onset [9].

This case report details a patient with recurrent episodes of GBS, focusing on the clinical presentation, diagnostic assessment, and therapeutic strategies. Thereafter, we shall discuss the current literature available on recurrent GBS and its clinical implications.

## Case presentation

Patient information

A 66-year-old lady, resident of Punjab and a homemaker with no known comorbidities, presented to the neurology clinic with complaints of gradual, progressive bilateral lower limb weakness followed by bilateral upper limb weakness, ongoing for 20 days before presentation. There was no history of numbness and paresthesia. There was no history of facial asymmetry, dysarthria, or loss of vision during the current admission. Her past medical history is significant for two similar episodes of limb weakness in 2012 and 2021. The first episode in 2012 was characterized by numbness of all four limbs along with difficulty in weight bearing, with subsequent frequent falls. The onset was acute and gradually progressive in nature over a course of 20 days. The second episode in 2021 was characterized by similar symptoms of numbness and weakness involving bilateral lower limbs for 10 days prior to presentation. There was no history of respiratory compromise during either episode. During both episodes, the patient returned to her baseline neurological status in the interim period, exhibiting no residual symptoms. There was no significant family history of malignancies or neurological disorders.

Clinical findings

During all three presentations, the patient was conscious and oriented to time, place, and person. Higher mental functions were intact, and no cranial nerve involvement was found in all three admissions. Motor power examination was variable during the different presentations, with proximal more than distal, lower limb more than upper limb weakness in all three presentations (Table 1). Deep tendon reflexes were absent in the lower limbs and diminished in the upper limbs with bilateral flexor plantar. There was no objective sensory involvement; however, the patient was ataxic, and tandem gait was noted to be impaired. Vibration and position sense were intact, indicating no deep sensory deficits.

**Table 1:** Comparison of muscle strength using the Medical Research Council (MRC) scale across episodes

Joint involved	Side	2012 power (MRC)	2021 power (MRC)	2025 power (MRC)
Shoulder flexors	Right	4/5	4/5	5/5
	Left	4/5	4/5	5/5
Shoulder extensors	Right	4/5	4/5	5/5
	Left	4/5	4/5	5/5
Shoulder abductors	Right	4/5	4+/5	4+/5
	Left	4/5	4/5	4+/5
Shoulder adductors	Right	4/5	4+/5	5/5
	Left	4/5	4/5	5/5
Elbow flexors	Right	4/5	4+/5	5/5
	Left	4/5	4+/5	5/5
Elbow extensors	Right	4/5	4/5	5/5
	Left	4/5	4/5	5/5
Wrist flexors	Right	4+/5	4/5	5/5
	Left	4+/5	4/5	5/5
Wrist extensors	Right	4/5	4/5	4/5
	Left	4/5	4/5	4/5
Hip flexors	Right	3/5	4/5	3+/5
	Left	3/5	4/5	3+/5
Hip extensors	Right	3/5	3/5	3+/5
	Left	3/5	3/5	3+/5
Knee flexors	Right	4/5	4-/5	3+/5
	Left	4/5	4-/5	4/5
Knee extensors	Right	4/5	4+/5	3+/5
	Left	4/5	4+/5	4/5
Ankle plantarflexors	Right	4/5	4+/5	4+/5
	Left	4/5	4+/5	3/5
Ankle dorsiflexors	Right	4/5	4+/5	4+/5
	Left	4/5	4+/5	4+/5

Diagnostic assessment

In the first episode in 2012, contrast-enhanced magnetic resonance imaging (CE-MRI) of the brain and cervical spine, as well as a nerve conduction study (NCS), was normal. However, cerebrospinal fluid (CSF) showed raised proteins suggestive of albuminocytological dissociation. A diagnosis of GBS (level two) was made as per the Brighton Collaboration criteria [10].

In the second episode in 2021, the NCS demonstrated prolonged distal latencies in bilateral median, peroneal, and tibial nerves with unobtainable H reflexes and median sensory responses, suggestive of a demyelinating type of neuropathy. CE-MRI brain with whole spine screening done showed nerve root enhancement at the level of cauda equina, which was likely suggestive of GBS. Apart from mild degenerative spondyloarthropathy in the cervical and lumbar spine, no significant neural foraminal narrowing or spinal canal stenosis was found. CSF analysis at the time was again suggestive of albuminocytological dissociation with normal cell count and elevated protein level.

**Figure 1:**
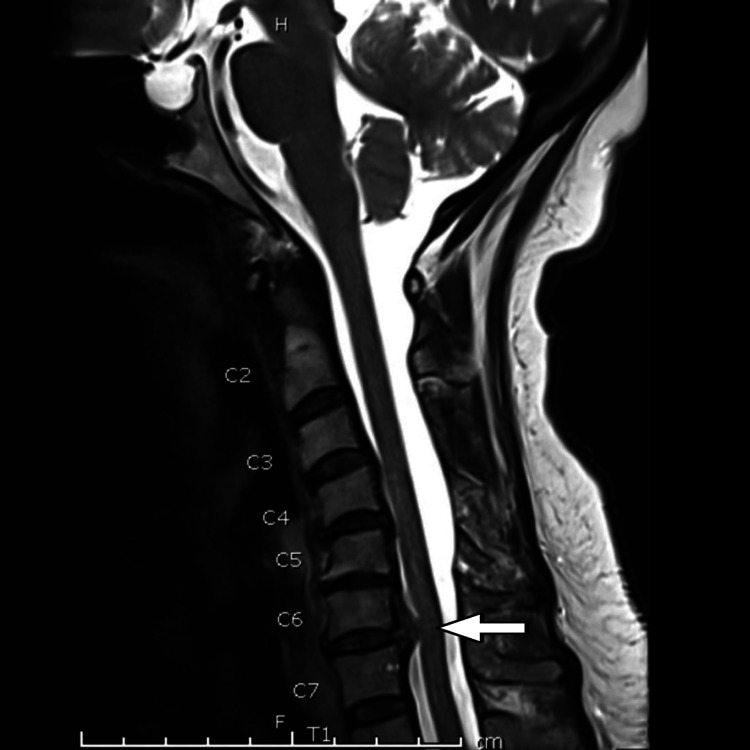
T2-weighted MRI of the cervical spine showing degenerative spondyloarthropathy at C6/C7 with spinal canal compression and cord indentation The arrow indicates a degenerative disc-osteophyte complex at the C6/C7 level causing spinal canal narrowing and indentation of the cord. Cord signal changes were absent. MRI: magnetic resonance imaging

In the current admission, the NCS again demonstrated demyelinating changes with prolonged distal latencies and low velocities. The NCS findings from 2021 and 2025 have been compared in Tables 2, 3. The NCS report from 2012 is not available for comparison. A cervical spine MRI was repeated in this admission due to a history of a recent fall, which showed degenerative spondyloarthropathy with osseous, discal, and facet changes involving the cervical spine predominantly at the C6/C7 level, causing spinal canal compression and indentation of the cord. No significant cord signal changes were found (Figure 1).

**Table 2:** Comparison of the motor nerve conduction study from 2021 and 2025 The above NCS illustrates a demyelinating type of neuropathy pattern characterized by significantly prolonged distal latencies and marked slowing of conduction velocities across the studies’ nerve segments (more in 2025 than 2021). DML: distal motor latency; CMAP: compound muscle action potential amplitude; MCV: motor conduction velocity; NCS: nerve conduction study

Presentation (year)	Nerve	DML (ms)	CMAP (mV)	F-response latency (ms)	MCV (m/s)
2021	Left median	8.4	5.2	29.8	53.1
2025		11.4	3.4	31.5	34.9
2021	Right median	10.5	4.1	30.1	47.9
2025		11	4.6	35.3	37.2
2021	Left ulnar	6.9	5.7	30.1	66.7
2025		7.7	4.8	32.4	51.3
2021	Right ulnar	7.2	5.7	29.1	57.1
2025		7.3	4.3	29.8	54.8
2021	Left peroneal	15.4	2.3	58.8	39.8
2025		14.8	2.5	59.4	35.9
2021	Right peroneal	14.4	3	47.1	42.9
2025		15.5	2.3	59.2	36.5
2021	Left tibial	14.9	5.3	49.8	43.5
2025		13.5	3.8	55.9	40.7
2021	Right tibial	13.7	2.9	49.8	43.7
2025		12.6	2.9	63.1	42

**Table 3:** Comparison of the sensory nerve conduction study from 2021 and 2025 SNAP: sensory nerve action potential

Presentation (year)	Nerve	SNAP (µV)	Onset latency (ms)	Peak latency (ms)
2021	Left median	20.1	2.8	4.2
2025		12	2.3	4.2
2021	Right median	21.7	3.5	4.9
2025		11.8	2.7	4.4
2021	Left ulnar	14	2.8	4.9
2025		19.9	4.7	7
2021	Right ulnar	10	2.9	4.6
2025		13.2	2.2	3.8
2021	Left sural	16	2.1	3.8
2025		27	1.9	3.7
2021	Right sural	23	2.4	4.3
2025		31	2	3.7

A ganglioside antibody profile was sent, which was positive for GM1(IgM) and GM3(IgM), and indeterminate for GM2(IgM), GD1a(IgM), and GQ1b(IgG). All other ganglioside antibodies were within normal limits. All biochemical and hematological parameters were found to be within normal limits. The patient was subsequently started on IVIG therapy.

Therapeutic intervention

The patient was treated with IVIG for both previous episodes, which was followed by a gradual recovery of symptoms. The patient achieved near-complete recovery within four months of physical rehabilitation. The current episode was also treated with IVIG, a total of 90 g over a period of three days. Physiotherapy was maintained daily throughout the course of her hospital stay. Supportive care in the form of intravenous fluids and supplementation was provided. The improvement in her symptoms was spontaneous with near-complete recovery in two months.

Follow-up and outcomes

**Figure 2:**
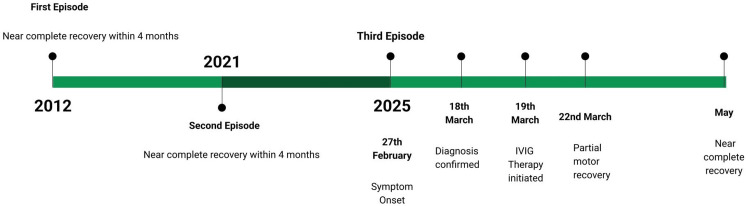
Timeline of the patient's clinical course Timeline of the patient's clinical course, delineating each episode of recurrence and the period of recovery. The timeline also highlights the symptom onset, diagnosis date, treatment intervention, and recovery milestones during the current (2025) episode. IVIG: intravenous immunoglobulin

The patient showed partial motor recovery at the time of discharge. The patient was advised to undergo regular physiotherapy, and exercises were taught by the neuro-physiotherapy team. At the two-week follow-up, the patient was found to have resolution of symptoms with persistence of some distal weakness in bilateral upper limbs. At the one-month follow-up, she was found to have near-complete recovery of power with minimal weakness in hand grip bilaterally. A summarized timeline of the patient's clinical course, delineating each episode of recurrence along with key events and interventions during the current episode, is presented in Figure 2.

Informed consent

Written informed consent was obtained from the patient for the publication of this case report.

## Discussion

The concept of recurrent GBS is still evolving, and several knowledge gaps persist [10]. It is hypothesized that infections, vaccinations, or other immunological stimuli can trigger aberrant immune system responses. The antibodies by the trigger-driven immune dysregulation further react with neural antigens, culminating in demyelination and axonal damage [1]. The second episode of GBS our patient experienced was preceded by two doses of COVID-19 vaccination in March and April of 2021. There have been other reports of GBS occurring after COVID-19 vaccination; however, the onset is typically one to 28 days after the first dose of the vaccine. Therefore, it can be postulated that this episode was not triggered by the vaccination [5,11,12]. Neither of the other two episodes had antecedent triggers.

Recognizing recurrent GBS and differentiating it from GBS-TRF or A-CIDP is difficult but essential because prognosis and treatment strategies significantly differ [13]. This distinction can be made by analyzing clinical timelines and utilizing electrophysiological studies [14]. Our patient's clinical presentation of acute, ascending weakness with areflexia and a monophasic course for each episode is characteristic of GBS rather than the chronic or relapsing course expected in CIDP. While albuminocytologic dissociation in CSF and demyelinating features on NCSs can be seen in both conditions, the temporal profile of rapid progression over days to weeks strongly favors GBS. Additionally, the positivity for antiganglioside antibodies, which are reported in approximately 30%-40% of GBS cases and are associated with specific subtypes like AMAN, provides further immunopathological support for a diagnosis of GBS. Taken together, the acute clinical evolution, supportive electrophysiological findings, CSF profile, and presence of antiganglioside antibodies reinforce the diagnosis of recurrent GBS rather than CIDP [11,15]. Antiganglioside antibody profiles sent during the current episode were positive for multiple markers. In terms of the association of seropositivity for antiganglioside antibodies in the first episode with subsequent recurrences, results from limited studies to date are conflicting [13,16].

The acute flare-ups of recurrent GBS are managed in the same way as initial occurrences, primarily utilizing IVIG or plasma exchange (PLEX), which remain the standard treatments [17]. Prompt treatment remains essential, as was validated by the significant clinical improvement after early initiation of IVIG in all the episodes. The unique therapeutic challenge takes form when the question of preventing further relapses is brought forward. While immunomodulatory prophylaxis is not routine, it may be evaluated for those who experience frequent relapses, depending on individual circumstances, although supporting evidence is scarce [18,19]. To gain a comprehensive overview, we have incorporated a comparative table that summarizes the clinical features, diagnostic modalities, treatment approaches, and time intervals between recurrences of the cases reported in the literature (Table 4).

**Table 4:** Literature review of recurrent Guillain-Barré syndrome cases with clinical and treatment profiles NCS: nerve conduction study; CSF analysis: cerebrospinal fluid analysis; IVIG: intravenous immunoglobulin; PLEX: plasma exchange

No.	Author	Year of publication	Gender	Episode	Age (years)	Diagnostic modality	Treatment	Time interval between recurrence
1	Imam and Liu [11]	2020	Male	1	31	NCS	PLEX and IVIG	12 years
				2	43	Ganglioside antibody test	PLEX	
2	Othman et al. [13]	2024	Female	1	63	Not mentioned	IVIG	17 years
				2	80	CSF analysis	PLEX and IVIG	
3	Bellucci et al. [12]	2022	Male	1	57	NCS, CSF analysis, ganglioside antibody test	IVIG	6 months
				2	57	NCS, CSF analysis, ganglioside antibody test	IVIG	
4	Kadam et al. [15]	2017	Female	1	9	Clinical diagnosis	IVIG	1 year
				2	10	NCS, CSF analysis	IVIG	
5	Gunatilake et al. [14]	2016	Female	1	13	NCS	IVIG	12 years
				2	25	NCS	IVIG	
6	Takemoto et al. [16]	2023	Male	1	19	NCS, ganglioside antibody test	IVIG	2 years
				2	21	NCS, ganglioside antibody test	PLEX and IVIG	
7	Takahashi et al. [19]	2018	Female	1	66	NCS	IVIG	13 years
				2	79	NCS	IVIG	

## Conclusions

Recurrent GBS is a rare but clinically significant entity. It specifically presents a diagnostic challenge due to its overlapping features with A-CIDP and GBS-TRF. This conundrum emphasizes the necessity of clinicians to develop awareness regarding the possibility of recurrence in individuals with a prior diagnosis of GBS. Continued research is necessary to understand the immunopathogenesis of recurrent GBS better and to evaluate the efficacy of immunomodulatory prophylaxis. Prompt treatment with IVIG and tailored treatment strategies remains fundamental in the management of such patients. Close monitoring, early relapse detection, and comprehensive rehabilitation support are also essential for reducing disability in recurrent cases and ensuring superior long-term outcomes. Ultimately, recurrence pattern recognition, immunological trigger identification, and prompt intervention may improve patient-specific prognostication and preventive strategies, contributing to clinical practice and scientific understanding of this complex neurological disorder.
